# Seroprevalence and Predictors of Hepatitis B Virus Infection among Pregnant Women Attending Routine Antenatal Care in Arba Minch Hospital, South Ethiopia

**DOI:** 10.1155/2016/9290163

**Published:** 2016-01-24

**Authors:** Tsegaye Yohanes, Zerihun Zerdo, Nega Chufamo

**Affiliations:** ^1^Department of Medical Laboratory Science, Arba Minch University, P.O. Box 21, Arba Minch, Ethiopia; ^2^School of Medicine, Department of Obstetrics and Gynecology, Arba Minch University, P.O. Box 21, Arba Minch, Ethiopia

## Abstract

Hepatitis B virus (HBV) is a serious cause of liver disease affecting millions of people throughout the world. When HBV is acquired during pregnancy, prenatal transmission can occur to the fetus. Therefore, this study is aimed at estimating seroprevalence and associated factors of HBV infection among pregnant women attending Antenatal Clinic (ANC) of Arba Minch Hospital, Southern Ethiopia. A facility based cross-sectional study was conducted on 232 pregnant women visiting ANC from February to April, 2015. Data regarding sociodemographic and associated factors were gathered using questionnaire. Serum samples were tested for hepatitis B surface antigen (HBsAg) by Enzyme Linked Immunosorbent Assay. Data was analyzed using SPSS version 20. The overall seroprevalence of HBV infection was 4.3% (95% CI: 2.2–6.9%). Multivariate analysis showed that history of abortion (AOR = 7.775; 95% CI: 1.538–39.301) and having multiple sexual partners (AOR = 7.189; 95% CI: 1.039–49.755) were independent predictors of HBsAg seropositivity. In conclusion, the prevalence of HBV infection is intermediate. Therefore, screening HBV infection should be routine part of ANC; health information on having single sexual partner for women of childbearing age and on following aseptic techniques during abortion should be provided to health facilities working on abortion.

## 1. Introduction

Hepatitis B virus is a potentially life-threatening cause of liver disease in the world. It both causes chronic infection and puts people at high risk of death from cirrhosis and liver cancer [1]. Globally, it is estimated that more than 2 billion people are still living with HBV infection. Over 350 million are believed to be chronically infected with the virus and are thought to be at a high risk of developing chronic hepatitis, cirrhosis, and primary hepatocellular carcinoma. About 1.2 million die annually from chronic hepatitis, cirrhosis, and hepatocellular carcinoma [2, 3].

Viral hepatitis during pregnancy is associated with high risk of maternal complications and high rate of vertical transmission. Fetal and neonatal hepatitis acquired from mother during pregnancy lead to impaired cognitive and physical development in latter life of the children [4]. The risk of vertical transmission depends on the time at which pregnant woman acquired HBV infection and on her statuses of HBsAg and hepatitis B early antigen (HBeAg) [5]. In the absence of immunoprophylaxis 10–20% of women seropositive for HBsAg transmit the virus to their neonates. Vertical transmission rate reaches approximately 90% when women are seropositive for both HBsAg and HBeAg [6].

The prevalence of chronic HBV infection is categorized as high (≥8%), intermediate (2–7%), and low (<2%) [7]. In developed countries, the incidence of hepatitis is around 0.1% whereas in developing countries it ranges from 3 to 20% and even higher in some areas [8]. In Africa and Asia, the prevalence of HBV is > 8% and 2 billion people have markers of current or past infection with HBV [9]. About half of new infections result from vertical transmission during pregnancy, a statistic that is linked to the fact that HBV screening is not part of routine antenatal care in the area [10].

Immunization is estimated to avert between 2 and 3 million deaths globally each year. In Ethiopia, routine immunization was launched in 1980. Ethiopia has successfully introduced hepatitis B vaccine in the form of pentavalent combination vaccine into the routine schedule in 2007. Children under the age of one year are the target group for the vaccination [11]. There is no widely available treatment for chronic hepatitis B and the other sequelae of HBV infection in the country, and if they are available, this treatment cost falls on the individual patient [12, 13].

In Ethiopia, the prevalence of liver disease is high and accounts for 12% of the hospital admissions and 31% of mortality rate [14]. The prevalence of HBV infection among pregnant mothers in Addis Ababa was 7% and 50% had evidence of infection at the age of 20 years [15]. In similar studies conducted among pregnant women in Jimma, the prevalence of HBV infection was 3.7% [16] while it was 4.9% and 8.1% among pregnant women in Dessie and Mekelle, respectively [17, 18].

Several studies around the world recommended that pregnant women should be screened for hepatitis B before delivery, as this offers an opportunity to prevent another generation from being chronically infected by the virus. However, in Ethiopia laboratory diagnosis of HBV infection is not part of routine care in ANC of all health facilities. Moreover, there is little information concerning seroprevalence of HBV infection among pregnant women and the existing data indicate that it differ from region to region as indicated above. Therefore, the present study is aimed to estimate seroprevalence of HBV infection and to identify associated risk factors among pregnant women attending ANC in Arba Minch Hospital, South Ethiopia.

## 2. Methods

### 2.1. Study Setting and Periods

The study was conducted from February to April 2015 in Antenatal Clinic of Arba Minch Hospital, which is found in Arba Minch Town. Arba Minch is located 505 kilometers in the southern part of Addis Ababa. The town is located in an altitude of 1200–1300 meters above sea level, with average annual temperature of 29°C. Arba Minch Hospital serves more than 2 million people in South Nations Nationalities and Peoples Regional State in Ethiopia.

### 2.2. Study Design and Sample Size

A facility based cross-sectional study design was used to enroll 232 pregnant women attending ANC in Arba Minch General Hospital. The sample size was determined using single population proportion formula to estimate the prevalence of HBV infection and* Toxoplasma gondii* infection among pregnant mothers attending ANC in Arba Minch General Hospital (larger project). The sample size is calculated based on the following assumptions: prevalence of HBV [18] and* T. gondii* [19] infections as 8.1% and 83.6%, respectively; 95% level of confidence; 5% margin of error. Finally, 10% of nonresponse rate was added to the calculated sample size. Accordingly the minimum calculated sample size for HBV and* T. gondii* infections was 126 and 232. Finally we took the larger sample size calculated (*n* = 232).

### 2.3. Study Population and Sampling Technique

The present study was conducted among pregnant women attending ANC in Arba Minch Hospital and mentally fit to respond the questions. Systematic sampling technique was used to recruit pregnant mothers in the study. A total of 696 pregnant women attended the ANC clinic during the past three months before study was initiated. This number was divided for the sample size to get the sample interval (*k* value) which is 3. Therefore, every 3rd mother attending the clinic was enrolled in the study until the calculated sample size was achieved within three months of data collection.

### 2.4. Data Collection

A pretested and structured questionnaire was used to collect information on sociodemographic, risky sexual behavior, history of hospital admission, history of abortion, and contact with HBV infected individuals. The data was collected through face-to-face interview by using nurses working in the ANC clinic of the hospital. Following the interview, approximately 2 mL of venous blood was collected from each consenting study participant by experienced phlebotomist. The blood was processed according to the standard operating procedures. Briefly, serum was separated from red blood cells and stored at −20°C prior to assay. Finally, the serum was tested for HBsAg using ELISA test kit (DIALAB) at Arba Minch National blood bank center laboratory, strictly following the manufacturer's instruction.

### 2.5. Data Analysis

After checking for completeness and consistency of the collected information, the data was entered into computer, cleaned, and analyzed using SPSS version 20.0 software package. Descriptive statistic was performed to describe demographic profile of the study participants. Bivariate and multivariate logistic regressions were used to assess the association between potential risk factors considered and HBV infection. Variables with *p* value < 0.25 by the bivariate analysis were entered into multivariate model. At multivariate logistic regression, *p* value < 0.05 was set as statistically significant for all variables.

### 2.6. Ethical Considerations

Ethical clearance was approved and obtained from Arba Minch University College of Medicine and Health Science Research Ethical Review Committee. Official permission was sought from Arba Minch Zonal Health Bureau and Arba Minch Hospital Administration. Moreover, written informed consent was obtained from all study participants prior to interview and blood collection. Confidentiality of the collected information and laboratory test results was maintained. Individual test results were communicated with the attending physician for further management of the cases.

## 3. Results

### 3.1. Sociodemographic Characteristics

A total of 232 pregnant women who had been attending Arba Minch Hospital ANC clinic from February 2015 to April 2015 were included in the study. The mean age of the participants was 25.98 years dominantly within the age range of 25–29 years and standard deviation (SD) was 4.49. Higher proportions of the subjects were married and urban dwellers. Majority of the participants were housewives and had studied up to primary level education (Table 1).

### 3.2. Seroprevalence and Associated Factors of HBV Infection

Of 232 pregnant women tested for HBsAg, 10 (4.3%) were found to be seropositive, giving the overall prevalence of 4.3% (95% CI: 2.2–6.9%). Pregnant women of age group of 25–29 years comprised 101 (43.5%) of the total study participants, of which 4 (4%) were seropositive. HBsAg seropositivity was observed to be higher in first trimester (5.1%) and the least in the second trimester (4.0%) but this difference was not statistically significant (*p* > 0.05). Moreover, none of the sociodemographic factors was significantly associated with HBsAg seropositivity (Table 2).

Sixteen (6.9%) of pregnant women attending ANC clinic had were reported to have history of blood transfusion and 2 (12.5%) of them were seropositive. However, blood transfusion was not significantly associated (*p* = 0.605) with prevalence of HBV infection among pregnant women attending ANC clinic in Arba Minch General Hospital. About 219 (94.4%) and 54 (23.3%) pregnant women had the practice of ear piercing and abortion, respectively. The respective prevalence of HBV infection among pregnant women who pierced their ear and aborted previously was 3.7% (*n* = 8) and 13% (*n* = 7). The significant association was observed between HBV infection and women who aborted previously (*p* = 0.013). Fifty-seven (24.6%) of the study participants had also a history of hospital admission and only 2 (3.5%) of them were seropositive. Concerning respondents of previous history of contracting Sexually Transmitted Disease (STD), of 10 pregnant women who had the disease, 2 (22.2%) were seropositive. The prevalence of HBsAg among women who suffered from STD was significantly higher than those who did not suffer from STD in univariate logistic regression (*p* = 0.020) but this association was not maintained after controlling the effect of confounding variables in multivariate logistic regression (*p* = 0.450). In this study, 12 (5.2%) pregnant women had previous history of multiple sexual partners and 2 (16.7%) of them were seropositive. Sexual practice with more than one individual significantly (*p* = 0.046) increases the transmission of HBV among pregnant women attending ANC clinic (Table 2).

## 4. Discussion

The seroprevalence of HBsAg among pregnant women attending ANC clinic in Arba Minch General Hospital was 4.3%. According to WHO classification, the prevalence of HBsAg was intermediate among pregnant mothers [20]. Unless preventive measures through vaccination are taken to tackle the risk of transmission, the unborn babies are at a higher risk of contracting HBV infection. The infection was significantly higher among pregnant mothers who had aborted previously and had history of sex with multiple sexual partners. Health facilities or organizations working on abortion of pregnancies play great role in the transmission of the virus which might be associated with using unsterile equipment during the abortion process. Moreover, unsafe sexual practice with multiple sexual partners is the major way of transmission of HBV among women of the childbearing age.

The prevalence of HBsAg among pregnant women attending ANC clinic in Arba Minch General Hospital was comparable with studies conducted in Bahir Dar (3.8%) [21], Jimma (3.7%) [16] and Dessie (4.9%) [17], Dares Salaam in Tanzania (3.9%) [22], and Lagos Nigeria (4.2%) [23]. In contrast to our study, the highest prevalence was reported from Benin (12.5%) [24], Cameron (10.2%) [25], and Mali (8%) [26]. On the other hand, the lowest prevalence was reported from two studies from India [27, 28]. The difference between the present studies and the above studies might be due to difference in socioeconomy and behavioral and cultural practices of age between 15 and 45 years.

In agreement with other studies [29, 30] HBsAg seropositivity was not significantly different by age. There was no difference in seropositivity of HBsAg between urban and rural pregnant mothers in the present study and it is in agreement with a study reported from Sana'a, Yemen [30]. In contrast to our study, study from Eastern Sudan has shown significantly higher prevalence of HBsAg among pregnant mothers from urban area than the rural counterparts [31]. This difference might be due to the varied numbers of urban and rural study participants as compared to our study.

According to this study, the prevalence of HBsAg was significantly higher among pregnant women who had history of abortion. Unplanned pregnancy is related to unprotected sexual intercourse which results in abortion and also increases the risk of HBV infection if such partners are infected. Also, instrumentation during abortion and related activities may serve as sources of exposure; all these circumstances may increase the likelihood of acquiring the infection. This is in agreement with a study reported from Lagos Nigeria [23] but in contrast with similar study carried out in North West of Iran [32].

In the current study, the rate of HBV infection was significantly higher and about seven times more likely to occur in those who had history of multiple sexual partners as compared to those who did not have multiple sexual partners. The significant association of having multiple sexual partners with HBV infection was also documented by other investigators [18, 33]. In contrast, some studies have shown that there was no significant association between abortion and seropositivity of HBsAg among pregnant mothers [22, 34]. Hepatitis B virus infection is sexually transmitted and the transmission increases with the duration of sexual activity and number of sexual partners [35, 36].

Blood transfusion is one of the means of transmission of bloodborne pathogen like HBV but it was not significantly associated with HBsAg seropositivity in the present study. This finding corroborates the report from Nigeria [37]. However, blood transfusion was significantly associated with transmission of HBV in a number of studies [21, 23, 28]. Improving the quality of laboratory screening of blood for HBV is one of the components in reducing the risk for transfusion-transmitted HBV. The possible explanation for absence of significant association between HBV infection and blood transfusion might be improved screening of HBV infection from blood donors before transfusion in Ethiopia.

The rate of vertical transmission of HBV infection is influenced by the time of pregnancy at which acute HBV infection occurs in the mothers [5]. In our study, the highest seroprevalence of HBV infection was found in those pregnant women at the first trimester as compared to second and third trimesters. However, there was no statistically significant difference in the prevalence of HBsAg seropositivity in different gestational age of pregnant mothers and it was in agreement with other studies [29, 38].

## 5. Conclusions

In conclusion, the prevalence of HBsAg among pregnant mothers attending ANC clinic in Arba Minch general hospital was intermediate. Abortion and unsafe sexual practice with more than one sexual partner play significant role in the transmission of the virus from infected person to healthy women of childbearing age. Therefore, screening pregnant women for HBV infection should be part of the routine care in ANC clinic in Arba Minch general hospital and in other similar settings. Health information on safe sex to women of childbearing age should be given to interrupt the transmission. In addition, health facilities working on abortion should strictly follow the aseptic techniques in order to save the life of both aborting mother and subsequent children of that mother.

## Figures and Tables

**Table 1:** Sociodemographic characteristics of pregnant women (*n* = 232) attending Antenatal Clinic at Arba Minch Hospital, 2015.

Sociodemographic characteristics	Count *N*	Percentage %
Age		
15–19	16	6.9%
20–24	69	29.7%
25–29	101	43.5%
30–34	35	15.1%
35–39	11	4.7%
Residence		
Urban	214	92.2%
Rural	18	7.8%
Marital status		
Married	224	96.6%
Single	6	2.6%
Divorced	0	0.0%
Widowed	2	0.9%
Educational status		
Unable to read and write	47	20.3%
Primary	79	34.1%
Secondary	71	30.6%
Tertiary	35	15.1%
Occupation		
Government	67	28.9%
Housewife	140	60.3%
Others^◊^	25	10.8%
Trimesters		
First (<14 weeks)	39	16.8%
Second (14–28 weeks)	124	53.4%
Third (>28 weeks)	69	29.7%
Gravidity		
Primigravidae	85	36.6%
Multigravidae	147	63.4%

◊ includes students, merchants, and farmers.

**Table 2:** Bivariate and multivariate analyses of factors associated with HBV infection in the study participants, Arba Minch Hospital, 2015.

Variables	Seroprevalence		COR (95% CI)	*p* value	AOR (95% CI)	*p* value
	Positive *n* (%)	Negative *n* (%)				
Age						
15–19	1 (6.2%)	15 (93.8%)	1.617 (0.169–15.458)	0.677		
20–24	3 (4.3%)	66 (95.7%)	1.102 (0.239–5.087)	0.901		
25–29	4 (4.0%)	97 (96.0%)	1			
30–34	1 (2.9%)	34 (97.1%)	0.713 (0.077–6.606)	0.766		
35–39	1 (9.1%)	10 (90.9%)	2.425 (0.247–23.850)	0.448		
Residence						
Urban	9 (4.2%)	205 (95.8%)	1			
Rural	1 (5.6%)	17 (94.4%)	1.340 (0.160–11.212)	0.787		
Educational status						
Unable to read and write	2 (4.3%)	45 (95.7%)	0.833 (0.147–4.734)	0.837		
Primary	4 (5.1%)	75 (94.9%)	1			
Secondary	2 (2.8%)	69 (97.2%)	0.543 (0.096–3.061)	0.489		
Tertiary	2 (5.7%)	33 (94.3%)	1.136 (0.198–6.514)	0.886		
Occupation						
Government	2 (3.0%)	65 (97.0%)	0.585 (0.118–2.893)	0.511		
Housewife	7 (5.0%)	133 (95.0%)	1			
Others	1 (4.0%)	24 (96.0%)	0.792 (0.093–6.728)	0.831		
Trimesters						
First (<14 weeks)	2 (5.1%)	37 (94.9%)	1.286 (0.240–6.908)	0.769		
Second (14–28 weeks)	5 (4.0%)	119 (96.0%)	1			
Third (>28 weeks)	3 (4.3%)	66 (95.7%)	1.082 (0.251–4.670)	0.916		
Gravidity						
Primigravidae	4 (4.7%)	81 (95.3%)	1.160 (0.318–4.234)	0.822		
Multigravidae	6 (4.1%)	141 (95.9%)	1			
Blood transfusion						
No	8 (3.7%)	208 (96.3%)	1		1	
Yes	2 (12.5%)	14 (87.5%)	3.714 (0.720–19.172)	0.117^⊕^	1.689 (0.232–12.296)	0.605
Ear piercing						
No	2 (15.4%)	11 (84.6%)	1		1	
Yes	8 (3.7%)	211 (96.3%)	0.209 (0.039–1.101)	0.065^⊕^	0.220 (0.022–2.161)	0.194
Nose piercing						
No	9 (4.1%)	213 (95.9%)	1			
Yes	1 (10.0%)	9 (90.0%)	2.630 (0.300–23.054)	0.383		
Body tattooing						
No	8 (3.7%)	209 (96.3%)	1		1	
Yes	2 (13.3%)	13 (86.7%)	4.019 (0.774–20.879)	0.098^⊕^	5.372 (0.617–46.751)	0.128
Tooth extraction						
No	8 (4.5%)	171 (95.5%)	1			
Yes	2 (3.8%)	51 (96.2%)	0.838 (0.173–4.073)	0.827		
Hospital admission						
No	8 (4.6%)	167 (95.4%)	1			
Yes	2 (3.5%)	55 (96.5%)	0.759 (0.156–3.682)	0.732		
History of surgery						
No	9 (4.2%)	204 (95.8%)	1			
Yes	1 (5.3%)	18 (94.7%)	1.259 (0.151–10.506)	0.831		
Contact with liver disease patient						
No	9 (4.1%)	211 (95.9%)	1			
Yes	1 (8.3%)	11 (91.7%)	2.131 (0.248–18.353)	0.491		
History of abortion						
No	3 (1.7%)	175 (98.3%)	1		1	
Yes	7 (13.0%)	47 (87.0%)	8.688 (2.163–34.891)	0.002^⊕^	7.775 (1.538–39.301)	0.013^*∗*^
History of alcohol drinking						
No	7 (3.6%)	187 (96.4%)	1		1	
Yes	3 (7.9%)	35 (92.1%)	3.562 (0.956–13.276)	0.058^⊕^	1.674 (0.339–8.257)	0.527
Delivery by TBA						
No	9 (4.2%)	204 (95.8%)	1			
Yes	1 (5.3%)	18 (94.7%)	1.259 (0.151–10.506)	0.831		
History of STD						
No	8 (3.6%)	215 (96.4%)	1		1	
Yes	2 (22.2%)	7 (77.8%)	7.679 (1.371–42.995)	0.020^⊕^	2.430 (0.243–24.295)	0.450
Multiple sexual partners						
No	8 (3.6%)	212 (96.4%)	1		1	
Yes	2 (16.7%)	10 (83.3%)	5.300 (0.993–28.275)	0.051^⊕^	7.189 (1.039–49.755)	0.046^*∗*^

^⊕^Candidate variable for multivariate analysis at *p* < 0.25;  ^*∗*^variable significant by the multivariate analysis at *p* < 0.05. COR: crude odds ratio, AOR: adjusted odds ratio, CI: confidence interval, TBA: traditional birth attendant, and STD: Sexually Transmitted Disease.
